# Critical Evaluation of Cross-Sectoral Collaborations to Inform the Implementation of the “One Health” Approach in Guadeloupe

**DOI:** 10.3389/fpubh.2021.652079

**Published:** 2021-08-02

**Authors:** Gaëlle Gruel, Mame Boucar Diouf, Catherine Abadie, Yolande Chilin-Charles, Eric Marcel Charles Etter, Mariana Geffroy, Cécile Herrmann Storck, Damien F. Meyer, Nonito Pagès, Gersende Pressat, Pierre-Yves Teycheney, Marie Umber, Anubis Vega-Rúa, Jennifer Pradel

**Affiliations:** ^1^Laboratory for the Study of Microbial Ecosystem Interactions, Institut Pasteur of Guadeloupe, Unit Transmission Reservoir and Pathogens Diversity, Les Abymes, France; ^2^INRAE, UR ASTRO, F-97170, Petit-Bourg, France; ^3^CIRAD, UMR AGAP Institut, F-97130, Capesterre Belle-Eau, France; ^4^AGAP Institut, Univ Montpellier, CIRAD, INRAE, Institut Agro, Montpellier, France; ^5^BGPI, Univ Montpellier, CIRAD, INRAE, Institut Agro, Montpellier, France; ^6^CIRAD, UMR BGPI, F-97130, Capesterre Belle-Eau, France; ^7^CIRAD, UMR ASTRE, F-97170, Petit-Bourg, France; ^8^ASTRE, Univ Montpellier, CIRAD INRAE, Montpellier, France; ^9^Centre Hospitalier Universitaire CHU de Guadeloupe, Laboratoire de Microbiologie Humaine et Environnementale, Les Abymes, France; ^10^Laboratory of Vector Control Research, Institut Pasteur of Guadeloupe, Unit Transmission Reservoir and Pathogens Diversity, Les Abymes, France

**Keywords:** One Health, evaluation, animal health, human health, plant health, environmental health, operationalization, interdisciplinary and cross-sectoral collaborations

## Abstract

In Guadeloupe, a French overseas territory located in the Eastern Caribbean, infectious and non-infectious diseases, loss of biodiversity, natural disasters and global change threaten the health and well-being of animals, plants, and people. Implementing the “One Health” (OH) approach is crucial to reduce the archipelago's vulnerability to these health threats. However, OH remains underdeveloped in Guadeloupe, hampering efficient and effective intersectoral and transdisciplinary collaborations for disease surveillance and control. A multidisciplinary research group of volunteer researchers working in Guadeloupe, with collective expertise in infectious diseases, undertook a study to identify key attributes for OH operationalization by reviewing past and current local collaborative health initiatives and analyzing how much they mobilized the OH framework. The research group developed and applied an operational OH framework to assess critically collaborative initiatives addressing local health issues. Based on a literature review, a set of 13 opinion-based key criteria was defined. The criteria and associated scoring were measured through semi-directed interviews guided by a questionnaire to critically evaluate four initiatives in animal, human, plant, and environmental health research and epidemiological surveillance. Gaps, levers, and prospects were identified that will help health communities in Guadeloupe envision how to implement the OH approach to better address local health challenges. The methodology is simple, generic, and pragmatic and relies on existing resources. It can be transposed and adapted to other contexts to improve effectiveness and efficiency of OH initiatives, based on lessons-learned of local past or current multi-interdisciplinary and intersectoral initiatives.

## Introduction

### Infectious Diseases Emergence and Wicked Health Problems

It is estimated that 60% of human emerging infectious diseases (EIDs) are zoonotic, of which more than 70–75% originate from wildlife (1, 2). This is exemplified by the emergence over the last 15 years of coronaviruses originating from animals, and more particularly of the SARS-CoV-2 virus causing the current COVID-19 pandemic (3–6). Global change, agricultural intensification, biodiversity loss, climate change, and wildlife trade are known to increase the frequency and incidence of EIDs. Emergence phenomena tend to increase over time (2, 7, 8), and ecosystem degradation is expected to intensify over the next decades (9), affecting local zoonotic host communities and creating hazardous interfaces between people, livestock, and wild reservoirs of zoonotic diseases resulting in increased pandemics risks (10). A panel of experts of the Intergovernmental Platform on Biodiversity and Ecosystem Services (IPBES) estimates that 631,000–827,000 of the 1.7 million undiscovered viruses existing in animals could have the ability to infect humans (11). Environmental pollutants are also known to promote metabolic disorders such as obesity, cardiovascular diseases, diabetes, cognitive development impairments, and cancers (12–14). The emergence and global spread of plant pathogens are also promoted by global change and trade, threatening food security, and human health (15, 16).

In this context, recommendations to implement multidisciplinary and cross-sectoral approaches to tackle complex health problems are increasing (11, 17–20), prompting efforts to address health issues at a global scale through “One Health” (OH) approaches in human, animal, and environmental sectors (7, 19, 21, 22). These approaches have the potential to improve the resilience of socio-ecosystems and reduce health disaster risks. Therefore, they are well-suited to vulnerable territories confronted by natural disasters, climate change, and health risks brought by international trade flows, such as Guadeloupe and other Caribbean islands (23).

### Problematic

The OH concept is defined as a collaborative, multisectoral, and transdisciplinary approach working at the local, regional, national, and global levels to achieve optimal health outcomes and recognizes the interconnection between people, animals, plants, and their shared environment (24). There is a consensus in the literature about the benefits of OH approaches such as improvements in human and animal health, well-being, and animal welfare, more effective and rapid disease control or biosecurity measures, improved information and data sharing, environmental protection for healthier ecosystems, enhanced social, and cultural values, more efficient disease surveillance networks (25). However, clear recommendations to successfully implement those approaches are critically needed (26–28).

### Rationale

OH remains underdeveloped and poorly promoted in Guadeloupe, impeding efficient and effective cross-sectoral and transdisciplinary collaborations to address the surveillance and control of zoonotic and plant diseases, or new emerging threats. In order to tackle those threats, new forms of collaboration involving multidisciplinary stakeholder groups from health and public/private sectors are needed. In this paper, a multidisciplinary research group reports on identifying key attributes for operational OH initiatives and their use to assess local animal, public, plant, and environmental health collaborative initiatives. The method is generic and can be adapted to other contexts to inform the implementation of an operational and impactful OH approach.

## Context

### Guadeloupe

Guadeloupe is a French overseas department located in the Lesser Antilles (Eastern Caribbean). Despite its modest size (1,628 km^2^), Guadeloupe archipelago concentrates a great diversity of land and marine ecosystems, making it one of the 34 world's biodiversity hotspots (9, 29). Guadeloupe is prone to natural disasters [hurricanes, earthquakes, volcanic eruptions, tsunamis (30)] and threatened by anthropogenic and climate change: habitat destruction, long-lasting water and soil contamination by persistent organic pollutants like pesticides (31–33), sea-level rise and increased frequency and intensity of extreme weather events (34). This results in emerging environmental health issues such as coral reef decline (35), Sahara sand dust (36), and harmful macroalgal blooms outbreaks causing massive strandings (37).

Guadeloupe has strong connections through tourism and trade with neighboring Caribbean countries and territories, North America, and Europe. This results in large flows of people, animals, plants, and by-products that threaten global health and local biodiversity, and agricultural productivity through the potential introduction of exotic pests and diseases. Thus, over the last two decades, Guadeloupe has experienced several emerging infectious disease outbreaks in humans or animals (West Nile virus, Chikungunya, Zika, dengue, leptospirosis, COVID-19) (38, 39), and crops (Black Leaf Streak Disease of banana, Huanglongbing of citrus, anthracnose of yam, coconut lethal yellowing.) (40–44). Additional exotic emerging infectious diseases, such as banana fusarium wilt tropical race 4 (45) or African swine fever (46, 47) are currently spreading worldwide at a worrying speed, hence threatening Guadeloupe's agriculture, economy, and food security.

To address these challenges, the effective OH implementation based on achievements of current/past programmes is crucial to commensurate with the challenges faced by the archipelago.

### Collaborative Research and Surveillance Programs in Guadeloupe

Guadeloupe has built strong local research communities and surveillance networks to characterize, prevent and control priority infectious diseases in humans, animals, and plants. This was done through a 6-year collaborative project, “Malin” (2014–2020) (48). The response to local health challenges relies on world-class scientific infrastructures, including reference, and high-level biosafety laboratories, with dedicated human resources. Public research organizations, hospitals, and agencies involved in human, animal, and plant health, surveillance, and innovation transfer engaged in interdisciplinary approaches and rationalization of resources via several collaborative initiatives. Four of them were assessed in this study:

- West Nile Virus (WNV) surveillance network. WNV is a mosquito-borne flavivirus that affects humans, equines, and birds. In 2002, seroconversions in horses and poultry provided the first indirect evidence of WNV circulation in Guadeloupe (49). Since then, epidemiological surveillance programs were enforced by several organizations involved in animal or public health to monitor WNV in horses, birds, mosquitoes, and humans and to improve knowledge on WNV epidemiology in Guadeloupe. However, the surveillance remains primarily sectoral with limited communication between its veterinary and public health components. After operating for more than 15 years, the network is currently shifting towards an integrated surveillance system with a pilot implementation of the OH approach (50).
- Black Leaf Streak Disease (BLSD) surveillance network. BLSD is a foliar disease of banana. It is caused by an ascomycete fungus causing major production losses (up to 100%) and reduced fruit greenlife, threatening the banana industry worldwide (51). The BLSD surveillance network was implemented in 2009 in Guadeloupe to prevent BLSD introduction. However, BLSD was first detected in Guadeloupe in 2012, prompting the network to promote a collaborative action plan including long-term disease management strategies targeting citizens and professionals from the banana industry. This plan included monthly biological surveillance based on the observation of sentinel banana plots spread over the entire territory. The network has since extended its activities to early detection and control of other banana emerging diseases.
- KaruBioNet is a collaborative interdisciplinary bioinformatics and biostatistics network. It was created in 2019 and involves scientists, engineers, and students. It aims to foster multidisciplinary collaborations and to provide mutual support for improving human, plant, and animal health in Guadeloupe through the implementation of bioinformatics. KaruBioNet members assist each other with the analysis, integration, and interpretation of bioinformatics data through shared access of the joint high-performance computing center of the University of Antilles (UA) (52).
- INSULA is a collaborative research project funded jointly by the European Commission and the Guadeloupe regional council. The project started in 2020 and aims to assess the influence of the ecosystem's biodiversity and its human-induced modifications on the diversity of vector-borne viruses affecting plants, animals, and humans in Guadeloupe, using the OH approach (53). It was implemented to bridge a collaboration gap between environmental health and ecology, building on a previous local collaboration between botanists and epidemiologists to unravel the eco-epidemiology of WNV in Guadeloupe (50, 54).

## Details to Understand Key Programmatic Elements

The development of an operational framework to assess how much local health initiatives have mobilized the OH approach has been conducted to identify major gaps, levers, and perspectives to enhance OH collaborations.

### Methods

Eighteen volunteer scientists referred to as “OH leaders” (OHLs) were involved in an 18-month capacity-building program on OH leadership. This program started in November 2019 and was facilitated by international experts: Profs. Craig Stephen (Canadian Wildlife Health Cooperation) and Christopher Oura (University of the West Indies). This OHL group includes researchers, engineers, Ph.D. students, laboratory technicians, medical/hospital, and epidemiological surveillance practitioners working in Guadeloupe in research/surveillance organizations. Their expertise encompass human, animal, and plant microbiology, medical entomology, animal and plant epidemiology, plant virology, and human infectious diseases. Volunteers joined this group following a call of interest launched within the Malin consortium. Motivation and commitment to follow the program were the only requirements for joining. The OHL group worked on this study between October and November 2020 as a part of the OH leadership program.

The OHL group searched for key attributes of successful OH operations. For this, members undertook a literature review of recent (<6 years) peer-reviewed publications on OH evaluation (55) published by OH reference groups like the Network of EcoHealth and OH (NEOH) (56) and organizations advocating for the implementation of OH internationally (57). They used PubMed and Web of Science, with One Health^*^, assessment^*^, implementation^*^, operational^*^, practice^*^, success^*^, recommendation^*^ and benefits^*^ as keywords. The OHL group shared publications using MoodleCloud^™^ and Mendeley^™^. Group members then reviewed, discussed, prioritized, and defined sets of criteria and their scores, corresponding to what the group judged critical for the successful implementation of OH initiatives, also using their own experiences. Consensual definitions for each Evaluation Criterion for OH Implementation (ECOHI) and their scoring rules were developed.

The OHL group developed a questionnaire to inform ECOHI scores using semi-directed group interviews (Supplementary Material). The questionnaire was first piloted on another collaborative health program (not assessed) conducted in Guadeloupe to evaluate its feasibility, then revised and reorganized to ease its utilization. Four local initiatives were selected for assessment based on the following criteria: they ought to be collaborative, multi/interdisciplinary and cross-sectoral, deal with animal, human, plant and/or environmental health, and ongoing (Table 1).

**Table 1:** Short description of the health collaborative initiatives assessed in Guadeloupe.

**Name**	**Nature**	**Health sector involved**	**Objective**	**Partners (disciplines and organization)**	**Other stakeholders involved**	**OH focused?**
WNV surveillance	Epidemiological surveillance network	Animal and human health	Monitor West Nile disease in sentinel animals and humans, improve WNV knowledge.	Medical practitioners, epidemiologists, hospital researchers (CHUG, CIRAD), public health practitioners (ARS, SPF), and agriculture organizations (DAAF), private veterinarians.	Horse center, poultry farmers	No
BLSD Surveillance	Epidemiological surveillance network	Plant Health	Prevent, monitor and control Black Leaf Streak Disease.	Scientists (CIRAD), government officers from the French plant protection services (DAAF), a technical organization specialized in plant health surveillance (FREDON), technical tropical crops Institute (IT2) and banana producers (LPG).	Family Garden	No
KaruBioNet	Expertise Network	Disciplinary network	Improve collaboration and structuration of bioinformatics in Guadeloupe.	Researchers, post-graduate students working in biology, computer science, health informatics, biostatistics, mathematics mainly. Partners: CIRAD, IPG, INSERM, INRAe, UA, CIC, Resource Biologic Center (KaruBioTec).	NA	No
INSULA	Research project	Animal, plant, human and environmental health	Assess the biodiversity of ecosystems and the influence of human-induced modifications on vector-borne viruses of plants, animals and humans risks.	Researchers in ecology, epidemiology, virology, entomology, botany, metagenomics from 4 research institutes in Guadeloupe (CIRAD, IPG, INRAe, UA) and Belgium (KU Leuven) as well as 2 NGOs involved in bird and bat conservation.	NGOs, government, regional, and international agencies involved in conservation, professional, citizen organizations, municipalities.	Yes

The questionnaire was used as a guide to assess the initiatives through interviews of groups of two to four persons most knowledgeable about each assessed initiative. Interviews intended to seek group perceptions and shared experiences between partners. Before interviews, each interviewee was informed about the goal and the course of the study. Interviewees accepted freely to participate, agreed that the interview was recorded and that the information shared would be anonymized and used for publication. All signed a formal letter of consent.

The four 2-h interviews were carried out between 19th and 25th November 2020 via ZOOM^™^. Interviewers were two OHLs familiar with the methodology but not with the assessed initiative. To minimize possible biases, an epidemiologist attended all interviews as an observer. The interviewers scored initiatives immediately after each interview. Scores were converted into percentages relative to the maximum ECOHI, and represented on a radar diagram using Excel software (Microsoft, Redmond, USA). During a final working session, the OHL group reviewed the results, harmonized their interpretation of the answers, and adjusted scores accordingly to minimize person-dependent variations. The individual criterion scores were averaged to compute the score of the initiative. A group brainstorming ensued to analyze and interpret the results in terms of gaps and successes and detailed recommendations to improve OH implementation.

A total of eight 3-h working sessions were organized by the OHL group, both face-to-face and virtually, using Microsoft TEAMS^™^.

### Results

A set of 13 opinion-based ECOHIs was developed and categorized in three types: “Governance”; “Partnership”; and “Resources” (Table 2), with scores ranging from 1 (minimum) to 2 to 4 (maximum) (Table 3).

**Table 2:** Selected criteria considered key attributes for the successful implementation of OH initiatives (ECOHI), definitions, and associated scoring.

**Category**	**#**	**Criterion**	**Definition**	**References**
1. Governance	1	Holistic thinking	The health problem is analyzed as a whole, using a systemic approach, considering the complex interactions between the processes and actors involved/concerned by health issues. The initiative has been thought of in a holistic, integrative, and non-specific way. It considers multiple disciplines, sectors (health and public/private), species, and integration at different spatial scales. It aims to integrate the knowledge of the various stakeholders, from the analysis of the problem to its resolution.	(58–60)
	2	Governance	New forms of governance to sustain relationships and long-term collaborations are defined (processes, rules) to ensure equitable distribution of decision-making power and resources. In addition, clear and transparent rules for operating principles and overall management exist.	(19, 28, 60–62)
	3	Collaborative planning	Planning requires that aims, problem formulation, responsibilities, and financing are clear, organized, and shared regardless of paradigms, organizational hierarchies, sectors, and stakeholders' disciplines. It needs resources (competencies, time, tools) to involve all key stakeholders in the planning; and clarity in establishing tasks and responsibilities.	(28, 59, 62)
	4	Adaptive coordination and monitoring	Due to the complex and cross-domain characteristics of OH collaboration, the initiative is deftly coordinated. It is characterized by adaptive planning and flexible implementation in the face of changes (new knowledge, the emergence of constraints or opportunities), making the initiative a part of a continuous improvement process. This dynamic monitoring of the initiative is characterized by the ability to continuously self-evaluate, learn, and adapt.	(27, 28, 59, 62, 63)
2. Partnership	5	Collaborative dimension and knowledge integration	The collaborative initiative involves stakeholders with different skills, working in public or private organizations (research, academia, producers, sales, public institutions, etc.) and health (animal, plant, environmental, and human) sectors in all phases (thinking, implementation, analysis, feedback). Participatory methods or a framework (multi-criteria analysis, system thinking, and transdisciplinary approach) are in place to engage stakeholders and integrate their knowledge (multi-criteria analysis, systemic analysis, transdisciplinary approaches, and other methodological guidelines).	(19, 27, 60, 63–66)
	6	Stakeholders diversity	A variety of stakeholders are involved in the initiative, including academic and non-academic groups, some of them traditionally linked to the health field (beneficiaries, ministries, international organizations, practitioners, technical institutes, industry) or not (private or public sector, other sectors of the civil society). They participate actively in the initiative, and they are effectively and ideally involved in all stages of the initiative.	(18, 26, 27, 63, 65)
	7	OH professionals' role recognition	One Health professionals have the freedom and ability to get involved in collaborative initiatives (by sharing their time, knowledge, skills, and other support). Their role is recognized and supported by their institutions/hierarchies and they can engage in horizontal programmes^*^. Mobility between sectors and organizations facilitates the development of “One Health” human resources. The recognition and support they receive marks an awareness of the “One Health” approach by their hierarchies and an understanding of its benefits. ^*^Horizontal programs are organized across institutions, teams, or services.	(65)
	8	OH awareness of non-scientific stakeholders	Non-technical and non-scientific stakeholders (donors, civil society, governmental/NGO organizations, and associations) are sensitized to the OH approach and take ownership of it, facilitating their participation in the initiative. This can result from active awareness campaigns (public debates, participatory workshops, training sessions, etc.) or other means of communication (press releases, website, social media, radio, T.V., etc.) organized by the initiative or by the stakeholders themselves.	(19, 27, 65)
	9	Soft skills of OH professionals	OH professionals of the initiative are trained on soft skills [participatory sciences, management (horizontal leadership), and communication (intercultural communication, conflict management)] to lead, operationalize and sustain OH programs. The technical skills needed to work in multidisciplinary settings, experience in group processes, and team development foster inter-professional communication, collaboration, and help build relationships and trust.	(28, 57, 61, 64, 67)
3. Resources	10	Supporting infrastructure	Supporting infrastructure (management tools, databases, human resources) is available to ease fund transfer between agencies and organizations to facilitate the implementation of OH programs. This enables monitoring and follow-up of multiple, strongly connected, and coordinated activities. It allows to more easily share (knowledge/information/resources, staff), learn from the initiative (knowledge exchange, institutional memory, feedback, self-regulation): and adopt a systemic organization (polycentric organization, high connectivity, synchronization, and multidimensions).	(28, 57, 61, 62, 64)
	11	Synergistic pooling of resources	Pooling of resources (human, financial, technical platforms, knowledge) beneficial to all parties is in place, enabling progress to be made on the initiative's critical points, organizing synergies, and optimizing these resources.	(19)
	12	Data and information sharing	Appropriate procedures for sharing and accessing data/information exist and are implemented. There are appropriate infrastructure and resources for managing heterogeneous data regarding quality, quantity, and nature. The willingness of stakeholders to share data and information is vital.	(18, 27, 28)
	13	Integrated data analysis	Data is collected following protocols defined and validated by the stakeholders. A data management plan has been put in place, facilitating the cleaning and validation of the data and its integrated analysis. This integrated analysis makes it possible to answer a common question and improve all partners' knowledge. All data from different partners is used in integrated data analysis.	(27, 64)

*ECOHIs were grouped into 3 categories: category 1, governance; category 2, partnership; category 3, resources*.

**Table 3:** Definition of the scores for each Evaluation Criteria for OH Implementation (ECOHI).

**ECOHI**	**Scoring levels**	**Scores' definitions**
1. Holistic thinking	3	1: Specific (sectoral/disciplinary) approach and analysis of the health problem were used. 2: A broader approach has been used to be more integrative of stakeholders (disciplines and sectors), however, there was no systemic analysis. 3: A holistic approach and a systemic analysis of the health problem were used.
2. Governance	2	1: There is no proper governance and rules and processes if they exist (decision making, operating principles, management) are not adapted. 2: There is good collaborative governance and coordination/information sharing mechanism aligned with rules and procedures.
3. Collaborative planning	3	1: Overall planning is organized according to sectors and organizational hierarchy. There is a lack of shared roles, responsibilities, and resources. There are no resources (competencies, time) to facilitate the initiative across sectors and disciplines. 2: Overall planning is organized regardless of sectors and organizations. Roles, responsibilities, and resources are shared however there are no/few resources to facilitate the initiative across sectors and disciplines. 3: Overall planning is organized regardless of sectors and organizations. Roles, responsibilities, and resources are shared and there are resources to facilitate the initiative across disciplines and sectors.
4. Adaptive coordination and monitoring	3	1: There is an annual monitoring process and basic coordination in place. 2: There are regular follow-up meetings with an analysis of difficulties/opportunities; however, no recommendations nor corrective/preventive actions are formulated/implemented. 3: There is dynamic monitoring and adaptive coordination of the initiative allowing evolving as changes occur. Recommendations or preventive and/or corrective actions are implemented.
5. Collaborative dimension and knowledge integration	4	1: The initiative is not collaborative: it is disciplinary and sectoral. 2: The collaborations are multidisciplinary but not multisectoral; there is no knowledge integration. 3: The collaborations are interdisciplinary and multisectoral, however, there is poor/some knowledge integration (no specific methods used). 4: The collaborations are inter/transdisciplinary and multisectoral, and stakeholders' partners knowledge is integrated using participatory or dedicated frameworks/methodologies.
6. Stakeholders' diversity	3	1: Stakeholders relevant to the initiative have not all been identified and do not participate in the initiative. 2: Stakeholders involved are only those traditionally associated with the health sector. They participate in all or part of the initiative. 3: Stakeholders including those associated with other sectors than health and relevant to the initiative have been identified and actively participate in all phases.
7. OH professionals' role recognition	3	1: The role of OH professionals is not recognized within their institution(s) and/or by the hierarchy. 2: The role of OH professionals is recognized, but they cannot invest time, share skills/knowledge, or provide any other support type in horizontal programs. 3: The role of OH professionals is recognized, allowing them to invest themselves in horizontal programs by sharing skills, knowledge, invest time, and provide any other type of support.
8. OH awareness of non-scientific stakeholders	3	1: Non-technical/scientific stakeholders are poorly informed/not aware of the OH approach used in the initiative. 2: Non-technical/scientific stakeholders are aware of the OH approach however they don't participate in the initiative. 3: Non-technical/scientific stakeholders take ownership of the OH approach and participate in the initiative.
9. Soft skills of OH professionals	3	1: No team building/trust development strategy is in place (awareness/training of stakeholders in humanities and behavioral sciences; organization of social events.). 2: A team-building/trust development strategy is in place (trained/awareness of stakeholders in humanities and behavioral sciences, social events organized as part of the initiative,.). 3: A team-building/trust development strategy is in place and is implemented to develop the social networking.
10. Supporting infrastructure	2	1: There is no supporting infrastructure other than the classical means of projects that are not multidisciplinary/sectoral. 2: Supporting infrastructure has been put into place and facilitates sharing, learning, and systemic organization.
11. Synergistic pooling of resources	3	1: No resource is available/allocated to the OH initiative; if resources are available, they are not pooled. 2: The available resources of the stakeholders are pooled for the OH initiative, but the benefits are limited to a couple of stakeholders. 3: Stakeholders' resources are pooled for the OH initiative and results in shared advantages/benefits with all parties.
12. Data/Information Sharing	3	1: No mechanism for sharing and managing data and information has been put in place and/or there is no willingness of data/information sharing. 2: There are procedures for data and information sharing and management, however, the access is restricted to a limited number of people or is not easy. 3: There is an active exchange of data and information between stakeholders following the procedures established within the initiative's framework.
13. Integrated data analysis	3	1: No definition of data collection protocol or data management plan. 2: A data collection protocol and/or data management plan has been developed, but the data analysis is not integrated. 3: A data collection and management are carried out as defined by the protocols and plans, the data analysis is integrated.

**Figure 1:**
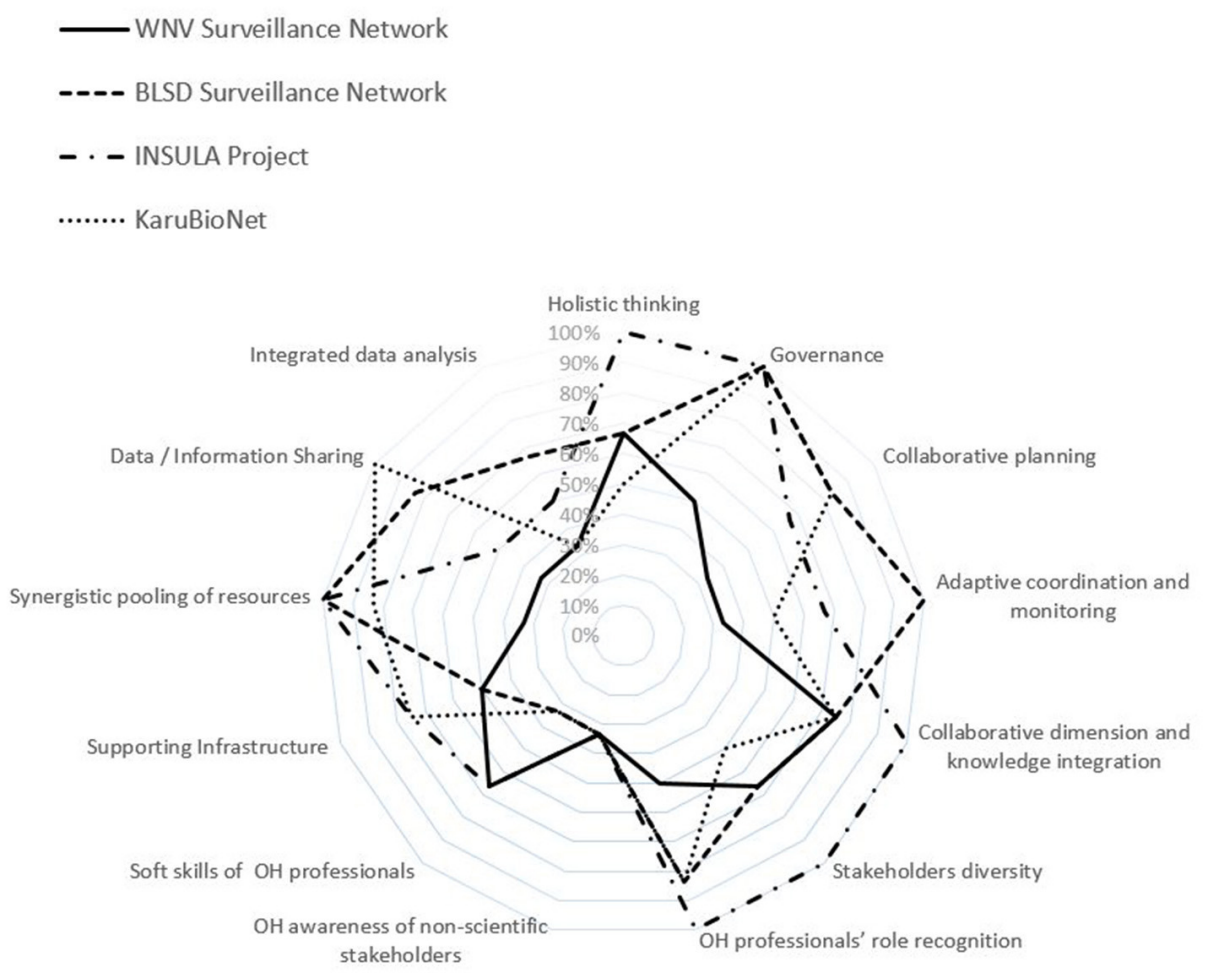
Radar diagrams displaying the scores (in %) for each ECOHI criterion for all the initiatives (Supplementary Figures 1A–D).

A total of 52 scores were obtained (Table 4). Figure 1 shows how assessed initiatives performed for each criterion according to their scores. The average score computed over the 13 ECOHI expresses the degree to which key OH attributes are applied. The variation of the scores may reflect differences in the nature and objectives of the initiative.

**Table 4:** Scores obtained for each criterion and each initiative, with a total score also expressed in relative percentage (in bracket).

	**ECOHI**	**WNV surveillance**	**BLSD surveillance**	**INSULA project**	**KaruBioNet**	**Score max**	**Average**
1	Holistic thinking	2 (67%)	2 (67%)	3 (100%)	1.5 (50%)	3	2.1 (71%)
2	Governance	1 (50%)	2 (100%)	2 (100%)	2 (100%)	2	1.7 (88%)
3	Collaborative planning	1 (33%)	2.5 (83%)	2 (67%)	2.5 (83%)	3	2 (67%)
4	Adaptive coordination and monitoring	1 (33%)	3 (100%)	2 (67%)	1.5 (50%)	3	1.9 (63%)
5	Collaborative dimension and integration of knowledge	3 (75%)	3 (75%)	4 (100%)	3 (75%)	4	3.2 (81%)
6	Diversity of the stakeholders involved	2 (67%)	2 (67%)	3 (100%)	1.5 (50%)	3	2.1 (71%)
7	OH professionals' role recognition	1.5 (50%)	2.5 (83%)	3 (100%)	2.5 (83%)	3	2.3 (79%)
8	OH awareness of non-scientific stakeholders	1 (33%)	1 (33%)	1 (33%)	1 (33%)	3	1 (33%)
9	Soft skills of OH professionals	2 (67%)	1 (33%)	2 (67%)	1 (33%)	3	1.5 (50%)
10	Supporting infrastructure	1 (50%)	1 (50%)	1.5 (75%)	1.5 (75%)	2	1.2 (63%)
11	Synergistic pooling of resources	1 (33%)	3 (100%)	3 (100%)	2.5 (83%)	3	2.4 (79%)
12	Data/Information sharing	1 (33%)	2.5 (83%)	1.5 (50%)	3 (100%)	3	2 (67%)
13	Integrated data analysis	1 (33%)	2 (67%)	1.5 (50%)	1 (33%)	3	1.38 (46%)
	Average program score (%)	18.5 (48%)	27.5 (72%)	29.5 (78%)	24.5 (65%)	38	25 (66%)

*Average scores were calculated for ECOHI and for each initiative*.

Overall, most initiatives performed well on some key attributes associated with interdisciplinary and cross-sectoral collaboration in health: “pooling of resources,” “collaborative dimension,” and “diversity of stakeholders involved” with some noticeable strength on “governance” and “recognition of the OH professionals' role.” On the contrary, they are weaker on “soft skills of OH workers,” “integrated data analysis,” and the “OH awareness of non-scientific/technical stakeholders” (Table 4).

The WNV surveillance network displayed the lowest overall score compared to the other initiatives, especially for ECOHIs of categories 1 (“collaborative planning” and “adaptive coordination and monitoring”) and 3 (“supporting infrastructure,” “pooling of resources,” “data/information sharing,” and “integrated data analysis”) (Table 4 and Supplementary Material). Some flaws in network governance (set of bodies and rules for decision-making, management, and operating principles to ensure strategic directions and oversight) negatively impact other ECOHIs such as “Integrated data analysis,” “data sharing,” or “synergistic pooling of resources.” Indeed, collaboration between the human and veterinary sectors in charge of equine, avian, and entomological surveillance remain low, despite WNV being an ideal OH disease model and the network being built using an integrative approach involving stakeholders from various disciplines in the animal and public health sectors willing to collaborate.

In contrast, the BLSD surveillance network had the second-highest average score and the best scores on several ECOHIs (Table 4 and Supplementary Material). The network has good governance and resource mobilization strategy (all resources available in surveillance partners were used for BLSD surveillance in a complementary way, with shared advantages/benefits for all parties) with rules ensuring equitable distribution of decision-making power and resources. This resulted in a high degree of collaboration between partners that translates into the high scores on “data/information sharing” (all partners received regular feedbacks) and “adaptive coordination and dynamic monitoring” (the network was highly flexible to adjust to changes in the disease situation). On the contrary, the network fared poorly for three ECOHIs: (i) “soft skills” (trust, team building, conflict management), that could help stakeholders to work better together if more complex problems arise in the future; (ii) “integrated data analysis” (only one partner in charge of data analysis); and (iii) “OH awareness” (OH is a new concept for most plant specialists). Finally, “supporting infrastructure” (management tools, databases, human resources) got a medium score: partners wished they could have more useful tools to save time for technical tasks.

The INSULA project had the highest average score (Table 4 and Supplementary Material). It is the only initiative that was conceived and implemented using a OH approach. Hence four critical ECOHIs of categories 1 and 2 reached maximum scores for “holistic thinking,” “new forms of governance,” “stakeholders' diversity,” and the “collaborative dimension and knowledge integration.” This scoring reflects the strong willingness of the project's partners (i) to implement an interdisciplinary approach; (ii) to involve ecologists and the environmental health sector in a health project; (iii) to focus on a cross-cutting topic, namely vector-borne viral diseases; and (iv) to share resources. Project partners have developed a common database and other collaborative tools for easier data/information sharing and integrated data analysis. Two ECOHIs could not be evaluated because no data had been produced yet at the time of interviews.

Despite being a relatively new network, KaruBioNet showed several assets (Table 4 and Supplementary Material). Its governance ensures equitable distribution of decision-making and resources; the pooling of resources and supporting infrastructure, including a shared super calculator made freely available for the local research community; sharing information, and data, which is the raison d'être of the network. The network was not initially conceived as a OH initiative; hence it fared poorly on several ECOHIs: “holistic thinking,” “adaptive coordination and monitoring,” and “stakeholders' diversity.” “OH awareness of non-scientific stakeholders” and “soft skills of OH professionals” were both not applicable. Interestingly, KaruBioNet does not conduct integrated data analysis as the information and data shared are not intended to be analyzed jointly. However, this network may do so in the future depending on its active involvement in collaborative projects and therefore become a key player in implementing the OH concept in Guadeloupe.

## Discussion

### Lessons-Learned From Current Collaborations

Challenges in implementing interdisciplinary and cross-sectoral programs occur at all stages throughout their lifespan (27). Interestingly, the framework developed in our study was applied to initiatives of different natures (research project, technical or disease surveillance networks) at different development stages—with WNV surveillance being the oldest (18 years old) and INSULA the most recent (5 months). The joint analysis of those initiatives, which share the same local context, provides relevant insights to inform future and ongoing collaborative OH initiatives in Guadeloupe. The OHL group also gained experience working on this joint study.

#### Study Highlights

##### Learning by Doing

The preliminary agreement on the meaning and definitions of ECOHI and scores greatly facilitated communication between the OHLs and with interviewees. Moreover, as previously experienced by similar groups (28, 68), the OHLs had to maximize organizational flexibility to overcome collaborative challenges. The teamwork's methodology and action plan were therefore revised at each group meeting to incorporate new insights and knowledge while balancing effective progress with members' commitment. This resulted in a more comprehensive program even though it took twice longer than planned.

All interviewees were positive about the study and acknowledged that it helped them change their perspective on OH. The semi-directed interviews allowed them to share experiences, to examine collectively past challenges and successes, and to reflect on recommendations for improving their own work. The method was simple, easily implemented, and delivered results quickly. The study also raised awareness on OH, of which most interviewees had only partial knowledge. Cross-sectoral communication benefited tremendously: e.g., it allowed animal health experts to exchange views on surveillance practices with plant health experts, and the OHLs were made aware of the initiatives evaluated throughout the evaluation process, which will help design future collaborative projects involving all health sectors.

Although three out of four initiatives were not initially OH in scope, they performed well on several key attributes associated with interdisciplinary and cross-sectoral collaboration. This encouraging result demonstrates that there is a local culture of collaboration. This could be explained by the small size of the territory, which favors the proximity of the different stakeholders of the scientific health community, exchanges and communication, making collaborations easier compared to larger territories.

##### Contrasting Results Between a Plant and a Zoonotic Disease Surveillance Network

Although WNV is a good model for developing OH approaches and despite a strong willingness of its members to collaborate, the WNV surveillance network scored low as the animal and human health sectors do not work together closely enough. On the contrary, the banana health surveillance network proved successful in delivering practical outcomes such as an efficient collaborative surveillance system based on early detection and an emergency response plan similar to what was successfully implemented in Australia (69).

The outcome of two decades of WNV sectorial work in Guadeloupe proved disappointing. The virus itself has not been isolated, its impact on human and animal populations is still unknown, and its local epidemiological cycle remains poorly understood. This situation could result from the epidemiology of WNV being complex in the Neotropics and very different from what is observed in North America (70, 71), and from the limited resources allocated to WNV in Guadeloupe. Since early 2020, WNV surveillance actors have been involved in a OH pilot project and agreed to create an integrated surveillance network aiming to operate more efficiently and effectively (50).

The BLSD initiative relies primarily on collaborations across disciplines and sectors (research, government agencies, technical institute, growers associations). This resulted in efficient management of the disease upon its outbreak in Guadeloupe and prevented panic in the population. The three network partners interviewed acknowledged that the high economic impact of BLSD helped achieve these outcomes because disease control was a top and shared priority for all stakeholders. This is a marked difference with the WNV network, as WNV is not a priority for the public health sector (50).

##### Continuous Improvement in the Implementation of the OH Concept in Guadeloupe

The INSULA project's maximum scores for several ECOHI (“holistic thinking,” “governance,” “collaborative dimension,” and “diversity of the stakeholders”) reflects progress in implementing OH in Guadeloupe. Indeed, the project benefits from previous experiences, such as the WNV and BLSD networks, through the direct participation of researchers involved in those networks. This helped avoid the pitfalls that have hampered earlier initiatives to various extent. In addition, the project was conceived and planned by a multidisciplinary group willing to collaborate with the environmental sector and tackle major environmental and health issues in Guadeloupe. Project participants agreed to share human resources, equipment, and infrastructures, demonstrating their willingness to move one step forward towards implementing the One Health approach in Guadeloupe. This has proved instrumental in securing funding from a competitive call for projects.

#### Gaps and Weaknesses

Overall, the evaluation conducted herein revealed that all assessed initiatives fare poorly on three ECOHI: 1/ “integrated data analysis”; 2/ “soft skills of OH professionals”; and 3/ “OH awareness of non-scientific stakeholders” showing that health communities in Guadeloupe still have to work on these aspects to foster the OH approach.

##### Integrated Data Analysis

“Integrated data analysis” is likely to improve in Guadeloupe if future projects are conceived collaboratively and if proper conditions and environments are created for data/information sharing (27). Although the INSULA project scored low for this criterion, it is likely to deliver a proper and shared integrated data analysis thanks to both project's governance and its design. Indeed, a consortium agreement is being prepared; dedicated secured platforms for data and information sharing are being created; protocols for data collection and management are being drafted. Finally, a dedicated 2-year engineer assistant has been recruited to collect, share, and manage data through a shared database under construction.

However, integrated data analysis can succeed only if challenges related to the implementation, monitoring and evaluation phases are anticipated and overcome (27, 28). These challenges include an ECOHI of extreme importance: “soft skills of OH professionals” for which the four initiatives fared poorly.

##### Importance of Soft Skills and Social Sciences

“Soft skills of OH professionals” include leadership, horizontal management, participatory sciences, experience in group processes, intercultural communication, conflict management, team development, etc. They are essential to lead, operationalize and sustain OH programs (28, 57, 61, 64, 67, 72), but are often overlooked (61). These skills can be brought by stakeholders and/or OH facilitators. They are instrumental in preventing and solving problems arising from collaborations between actors working in multiple domains, who do not have a collaboration history, or from institutional/academic or geographic and cultural fragmentation (27). Those skills are needed to work in multidisciplinary settings, foster communication, and relationship-building, which are essential in the local context, where the health and research systems are fragmented into small and scattered disciplinary teams. Development of trust and engagement of actors result in well-managed and coordinated collaborative programs with real integration of expertise and knowledge as opposed to artificial collaborations where stakeholders work together but remain in their silo (27); this was somehow experienced during the “Malin” project. If health communities of Guadeloupe aim to steward ambitious and long-lasting OH programs, they will need to define a strategy to fill those gaps in OH soft skills. In particular, collaboration with social scientists is important and efforts are ongoing.

##### Importance of OH Awareness

The lack of “OH awareness among non-technical and non-scientific stakeholders' groups” (e.g., funder, civil society, governmental/non-governmental organization, etc.) may have had a minor impact on the initiatives that did not have a strong OH scope. On the opposite, it is important to fill this gap with the many stakeholders of the INSULA project while it is getting off the ground, and with the WNV surveillance network that aims to shift towards an integrated network. According to their objectives and long-term goals, OH programs are expected to benefit tremendously from raising OH awareness among groups not traditionally involved in health projects. For instance, the BLSD surveillance network actors stressed out the importance of associating the public to the prevention and control of BLSD. Public awareness and actors' engagement maximize the impact of projects, promote innovations (65), and positively influence funding policies. It also enhances the proximity with scientists, thus contributing to fight the growing distrust of science among the public that prevents society from serenely debating major issues such as GMOs, vaccination or climate change (73).

##### Importance of Supporting Infrastructures

While supporting infrastructures (management tools, databases, human resources) are available for KaruBioNet and INSULA, they are vitally needed for the surveillance networks assessed. The lack of supporting infrastructures did not prevent the BLSD network from meeting its objectives, but it resulted in an increased workload for the actors, which is unsustainable in the long-term. This lack could prove problematic if another emerging banana disease was introduced and required additional work. In contrast, the lack of supporting infrastructure impacted WNV surveillance markedly, preventing the network from sharing data and information. An Information System coupled with RShiny (RStudio®) for dynamic and interactive data visualization was developed recently to pilot a more integrated network, along with new communication routes (50). More generally, surveillance networks would benefit from project management tools and geographic information systems to monitor the progress of control actions to support health interventions, reduce costs, and save time and energy.

##### Importance of Holistic Approach

Only one initiative (INSULA) scored maximum on “Holistic thinking,” meaning that a holistic approach and a systemic analysis of the health problem were used (Table 3), stressing the need for capacity-building in system thinking and system analysis in Guadeloupe, which can be conducted using participatory methods as described by Duboz et al. (58).

### Methodological Limitations

We proposed a semi-quantitative method based on the analysis of 13 criteria to assess the OH framework implementation in collaborative initiatives rapidly, whereas some participatory methods were developed to implement OH initiatives, such as disease surveillance, requiring several workshops and more time (74). If our approach delivers on results quickly, an action plan tailored to each initiative should be defined to improve its efficiency and effectiveness.

The design of interviews (different interviewers for each initiative assessed, some OHLs being interviewed) may generate biases. These biases were minimized by implementing corrective actions such as: group interviews, interviewers external to the initiative, same observing epidemiologist participating in all interviews, group analysis of the interviews results. No major difference was noticed in the interpretation of questions among the OHL group. Finally, emphasizing the main objective of the interviews—learning from initiatives rather than comparing their performance—helped keep objectivity. This design facilitated cross-sectoral communication and the exchange of experience.

Several excellent scores were assigned, suggesting an advanced level of key OH attributes implementation in Guadeloupe, although the OH approach remains under-developed in Guadeloupe. This is due to the limited number of scoring levels with maximum values accounting for both promising/good and excellent/outstanding results. Adding an additional scoring level would not have been relevant in our context. NEOH tools should be considered for a more thorough “OH-ness” assessment of more advanced OH programs (59).

Although the INSULA project is just starting, all ECOHIs were scored—those not applicable were scored minimum. In contrast, those relating to the implementation phase (“collaborative planning,” “adaptive coordination and monitoring,” “data sharing,” “integrated data surveillance”) were scored according to available information. The low scores reflect the lack of information rather than real issues. In general, it would be worth re-evaluating young initiatives like INSULA once they are more advanced.

### Implementing a Change-Oriented Strategy to Enhance OH in Guadeloupe

Identifying problems, gaps, and making recommendations is far easier than identifying implementable solutions leading to meaningful results (27). The work reported in this paper shows that the OH community in Guadeloupe is ready to move one step further towards the building of a strategy based on the theory of change to implement sustainable good OH practices involving diverse stakeholders.

For this, the OHL group will use an approach for building *ex-ante* impact pathways (“ImpresS *ex ante*”) based on the approach developed by Blundo Canto et al. (75). This participatory, iterative and adaptive approach is particularly well-suited to OH issues. It consists of a 3 to 4-day face-to-face participatory workshop. A group of relevant stakeholders, including decision makers, builds a shared vision of the future (desired impacts) over a 5 to 10-year period and develops a common strategy, including a plausible and sound implementation plan. Participants agree on desired outcomes regarding change of behavior, practices and capacities. Then, they identify the key challenges preventing those from occurring and propose plausible and realistic solutions that will overcome those challenges. This approach is being increasingly implemented to improve the impact of collaborative projects through easier, more efficient, and more fruitful collaborations. It has also been applied to strengthen stakeholders engagement and cooperation in surveillance systems to better tackle major challenges in public health such as antimicrobial resistance (74).

## Conclusions

The lessons learned from this study and the use of the methodological framework described in this paper are expected to improve not only existing initiatives but also the design and implementation of future ones. For example, the OHL group is currently building a new collaborative project based on a systemic analysis of the health problems they want to address, using the lessons learned from the Malin project, and the outcomes of this study.

The scope of our study can be improved and broadened by including more socio-economic analysis and programs carried out in Guadeloupe by other research groups and involving grass-roots or other stakeholders. The proposed strategy could also be adapted to other Caribbean states and territories, and be helpful for evaluating quickly OH collaborative initiatives around the world before more in-depth analysis.

As described in this paper, implementing OH approaches requires a paradigm shift towards fully effective, strategic and broad-spectrum institutional collaboration to ensure better health for humans, animals, plants, and the environment. This process can be viewed as the “Rosetta stone” that enables cross-sectoral associations to implement technical, organizational, and political solutions to address future health crises. We are confident that the synergy resulting from implementing a OH approach in Guadeloupe will help reshape its health system towards a more holistic health approach.

## Data Availability Statement

The raw data supporting the conclusions of this article will be made available by the authors, without undue reservation.

## Ethics Statement

Ethical review and approval was not required for the study on human participants in accordance with the local legislation and institutional requirements. The patients/participants provided their written informed consent to participate in this study.

## Author Contributions

JP designed, coordinated, and organized all phases of the project. JP, MG, CA, YC-C, P-YT, MD, MU, and CH conducted the interviews and data collection from partners. GG, MD, and JP drafted the article and supervised the writing of the paper. All authors participated in all steps of the work, contributed to the drafting and the revision of the article, and approved the submitted version.

## Conflict of Interest

The authors declare that the research was conducted in the absence of any commercial or financial relationships that could be construed as a potential conflict of interest.

## Publisher's Note

All claims expressed in this article are solely those of the authors and do not necessarily represent those of their affiliated organizations, or those of the publisher, the editors and the reviewers. Any product that may be evaluated in this article, or claim that may be made by its manufacturer, is not guaranteed or endorsed by the publisher.

## Abbreviations

ARS: Regional Health Agency
BLSD: Black Leaf Streak Disease
CHUG: University Hospital Center of Guadeloupe
CIRAD: French Agricultural Research Centre for International Development
DAAF: Food, Agriculture and Forest Direction of the ministry of agriculture
ECOHI: Evaluation Criteria for OH Implementation
EID: Emerging Infectious Diseases
ERDF: European Research and Development Funds
INRAe: National Research Institute for Agriculture Food and Environment
IPG: Institut Pasteur of Guadeloupe
MALIN: Maladies infectieuses en milieu insulaire tropical—infectious diseases in tropical island environments
NEOH: Network of EcoHealth and OH
SPF: Public Health France
WNV: West Nile Virus
UA: University of Antilles.
