# Supplementary material for: Critical Evaluation of Cross-Sectoral Collaborations to Inform the Implementation of the “One Health” Approach in Guadeloupe
Source: Front Public Health. 2021 Aug 2;9:652079. doi: 10.3389/fpubh.2021.652079 (PMC8366749; doi:10.3389/fpubh.2021.652079)
Supplement: Supplementary file 1 [file Data_Sheet_1.docx]

**Suplementary material – questionnaire**

**Part 1 - Presentation of the initiative to the interviewees**

**Background information on the Study**

Interdisciplinary and multisectoral collaborations to work more effectively on complex health issues using the "One Health" concept are theoretically ideal and have many advantages over sectoral approaches. In practice, they are difficult to set up and are therefore rarely or only partially implemented.

Within the framework of the “Malin” project, a team of 15 people from several research institutes (CIRAD, INRAE, IPG, CHU) trained on "One Health" leadership is in charge of strengthening collaborations between the four health sectors (human, animal, environmental and plant) with the shared objective of building and implementing future collaborative and ambitious "Health" projects in Guadeloupe.

The team guided by international experts Craig Stephen (Canadian Wildlife Health Cooperation) and Christopher Oura (University of the West Indies), conducted a literature review and drew on the experiences of its members to identify 13 key criteria for operational success of these approaches.

The team is now conducting a critical analysis of four emblematic collaborative initiatives conducted in Guadeloupe: a network of expertise in bioinformatics, two 2 epidemiological surveillance networks in plant health and animal/human health and the construction of a global health project. Objective: to see how each initiative mobilises the “One health” approach according to these 13 criteria, and to study more specifically the successes and failures of the implementation of interdisciplinary and multisectorial collaborations in order to draw recommendations to improve the effectiveness and impact of future global health projects in Guadeloupe.

This study is based on feedback through interviews involving 2 or 3 people per initiative. These people must be familiar with the initiative and have an overall view. The team conducting the interviews is particularly interested in the difficulties linked to the implementation of these complex collaborations and the solutions/ideas to overcome them.

**Organisation of the discussion**

- About 45 questions divided in 4 categories. Total duration of the interviews: about 2h
- Round table + explanations of the two interviewers for the administration of the questionnaire.
- Remind basic advice for a easy virtual communication (avoid simultaneous discussion, repeat in case of internet break, ensure that everyone expressed their opinion…)
- Take the time to explain new concepts / give clear definitions to technical terms
- Encourage interviewee to ask to repeat or ask clarification if needed
- Remind that interviews are not evaluations; therefore there are no right or wrong answers.
- Interest of the collective interviews is to complete the visions. There can be consensus or marked differences: every input is good. Everyone will be able to express their point of view.
- The interview is recorded. Only the persons involved in the study will access the information. The information provided will be used for the study. The results will be shared to check the information recorded. The results and the article will be shared to validate the information before submission.

**Concluding remarks and follow-up**

- Questionnaires from all 4 initiatives will be analysed jointly to draw general recommendations (capitalisation and feedback, sharing of ideas, etc.).
- A publication will be prepared on the basis of this work and it will be shared with interviewees upon acceptance
- The objectives of these questionnaires are to improve collaborative projects, to tackle more ambitious, more complex health issues.

**Part II – Questionnaire on the collaborative initiatives conducted in Guadeloupe**

**GENERAL KNOWLEDGE OF THE INITIATIVE**

1. Can you explain very briefly the health issue your initiative is about?
2. What is the objective of your initiative (in a few words)?
3. List all stakeholders* involved in the initiative and specify their disciplines** and sectors of activity***
4. Were (are) stakeholders actively involved in all or some phases of the initiative**** (specify which ones)?
5. Alternatively, specify which stakeholders were involved and in what phase(s) of the initiative.

* Stakeholders: beneficiaries, project partners, actors, …

** Disciplines/expertise: microbiology, virology, bioinformatics, entomology, epidemiology, ornithology, human and veterinary medicine, ecology, health management, project management, animal production, plant production, ....

*** Health sectors: animal, human, environmental, plant, etc. // Public/private sectors: research, education, producers, industry, public institutions, NGOs, etc.

**** Phases of the initiative: set-up, implementation and coordination, feedback, evaluation

**CATEGORY 1 - GOVERNANCE, SYSTEM THINKING, MANAGEMENT METHODS**

***ECOHI #1 - Holistic thinking and systemic analysis of health problems***

1. Was the initiative conceived in a holistic manner - i.e. taking the targeted health issue(s) as a whole, considering all the species involved in the epidemiological cycle, all the main factors known to influence the targeted health issue(s)?
2. Was a comprehensive problem analysis carried out to define the problem as a whole before preparing the initiative?

If yes: explain the method used in a few words

1. When identifying the partnership/consortium and stakeholders relevant to the initiative (beneficiaries, groups affected by / interested in the issue), did you consider:

i) different health sectors?

ii) different public/private sectors?

iii) different disciplines ?

***ECOHI #2 – New forms of governance***

1. In terms of governance - are there rules/procedures (charter, consortium agreement, other...) that explain how decision making, management and general operation is done?
2. Does the governance ensure that stakeholders are fairly involved in decision-making?

***ECOHI #3 – Collaborative planning***

1. How is work planning done in general? Is it done independently of organizations, hierarchies and/or sectors?
2. Have the roles and responsibilities of stakeholders been clearly established and allocated?
3. If so, how? (steering committee, others....)
4. Are there resources (skills*, time, tools) to facilitate the initiative and in particular to link sectors and disciplines? * Skills refer to facilitation, communication and leadership - or previous training in transdisciplinary methods

***ECOHI #4 – Agile coordination and dynamic monitoring of the initiative***

1. Describe the monitoring and evaluation method used for your initiative (organization, frequency/regularity, purpose of meetings, other modalities).
2. In case of constraints, challenges, opportunities and new knowledge - are there any adaptations, recommendations, corrective actions, program changes, new operating procedures...?
3. Are/were recommendations, new procedures translated into concrete actions within the framework of the initiative?

**CATEGORY 2 - PARTNERSHIP, COLLABORATION, SKILLS**

***ECOHI #5 – Dimension of collaboration and knowledge integration***

1. How would you describe collaboration between disciplines?
   1. Disciplinary: remains within the boundaries of one discipline or expert perspective
   2. Multidisciplinary: involves to some extent collaborations between different disciplines, without major integration of different forms of expertise and knowledge
   3. Interdisciplinary: involves a coordinated effort of collaboration and integration between disciplines
   4. Transdisciplinary: is about collaboration and integration between academic and non-academic groups such as scientists on the one hand and stakeholder groups, policy experts, the private sector or other practitioners on the other hand.
2. Explain how stakeholder knowledge is integrated* into the initiative? (participatory methods, multi-criteria analysis, systemic analysis, other?) *Knowledge integration: using stakeholder knowledge to develop shared syntheses to build a common framework for understanding the links between the knowledge of many individuals.

***ECOHI #6 – Diversity of stakeholders involved***

1. Have you identified stakeholders who are not traditionally associated with health initiatives (civil society, NGOs, public/private sector, ...) and who are relevant to your initiative?
   If yes, which ones? Name organizations / sector, indicate the type of stakeholders (beneficiary, concerned, actor, partner, ...)?

***ECOHI #7 – Recognition of the role of "One Health" professionals***

* A "One Health" professional is a person who is involved in a collaborative health initiative (not necessarily a leader)

1. Is the investment of professionals in the collaborative initiative supported/encouraged by their institute?
2. Did "One Health" professionals have the necessary capacity/resources available to invest in horizontal programs*? *Horizontal programs are organized between institutions, teams or departments
3. Does your institute/organization recognize the importance of the One Health concept and its potential advantages/benefits? (Name the institute)

***ECOHI #8 – Raising awareness of non-scientific/technical stakeholders about the "One Health" approach used in the project***

1. Has the initiative developed and implemented a strategy to raise awareness about the One Health approach among non-scientific/technical stakeholders?
   If so, what is the strategy used? Active awareness raising campaigns (public debates, participatory workshops, training sessions, ...) or other means of communication (press, media, website, social networks, radio/TV, ...) organized by the initiative or the stakeholders themselves.
2. How are non-technical/scientific stakeholders involved in the initiative? What is their input?

***ECOHI #9 – Training in social and behavioural sciences for "One Health" professionals***

1. Is there strategy to enhance team-building and confidence-building (such as organisation of social events, training/awareness in social and behavioural human sciences*) *Participatory sciences, management (leadership, transversal management) and communication (conflict management, participatory sciences, intercultural communication)...
2. If so, has this been / is it being put into practice?
3. Do you organise outings/social events in the framework of the initiative?
4. Did/does this help strengthening the network of partners?

**CATEGORY 3 - RESOURCES, TOOLS, SUPPORT INFRASTRUCTURES**

***ECOHI #10 – Support infrastructures***

1. Would you say that the support infrastructures* consist in the classic means used in project management, not necessarily multidisciplinary or multisectoral projects?

*Support infrastructures (management tools, databases, human resources, IT support, laboratories, other structures...) including to facilitate the transfer of funds between agencies/organizations

1. What is being done in the initiative to facilitate:
   1. sharing (of knowledge, information, resources, staff);
   2. learning (knowledge exchange, institutional memory, feedback, self-regulation);
   3. adoption of a systemic organization (polycentric, with high connectivity, synchronous and multi-dimensional actions)?

***ECOHI #11 – Synergistic pooling of resources***

1. Have resources (human, financial, equipment, premises, knowledge, etc.) been allocated to the initiative?
2. Have these resources been pooled/used effectively by several actors in the initiative? If so, which ones?
3. Was the pooling of these resources beneficial to all parties

***ECOHI #12 – Data and information sharing***

1. Have procedures for sharing data (any type) and information been considered or implemented to carry out this initiative? If so, which ones?
2. Have resources (human, financial, tools..) been allocated to facilitate and ensure data/information sharing (databases)?
3. Is the exchange of data and information smooth?

***ECOHI #13 – Integrated data analysis***

1. Is there a data collection protocol and/or data management plan in place for the initiative?
2. How is the data from the initiative analyzed?
3. Who is involved on data analysis?

**FINAL QUESTIONS**

1. What do you think was the biggest challenge – and what was (would need to be) done to overcome it?
2. What was the biggest success of your initiative – and what are according to you the main factors for success?
3. If there was only one thing that could be done to significantly improve collaboration, what would it be according to each of you?
4. What do you think of the work done with you today: is it useful for you? For your initiative?
