# Supplementary figures and images for: Critical Evaluation of Cross-Sectoral Collaborations to Inform the Implementation of the “One Health” Approach in Guadeloupe
Source: Front Public Health. 2021 Aug 2;9:652079. doi: 10.3389/fpubh.2021.652079 (PMC8366749; doi:10.3389/fpubh.2021.652079)

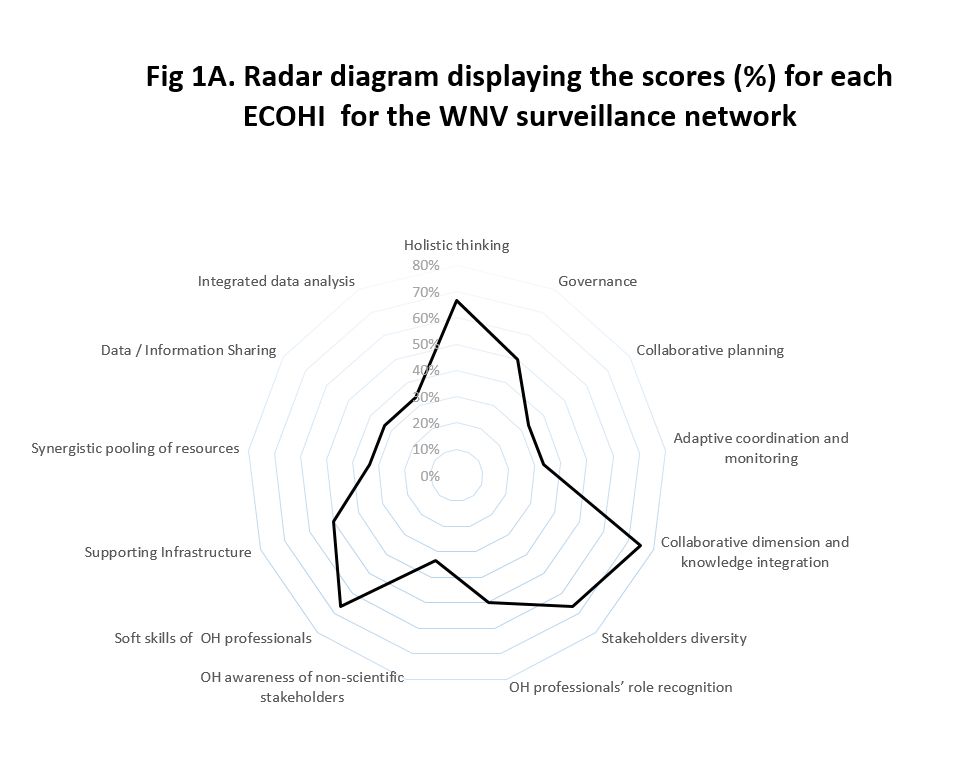

Supplement: Supplementary file 2 [file Image_1.jpg]

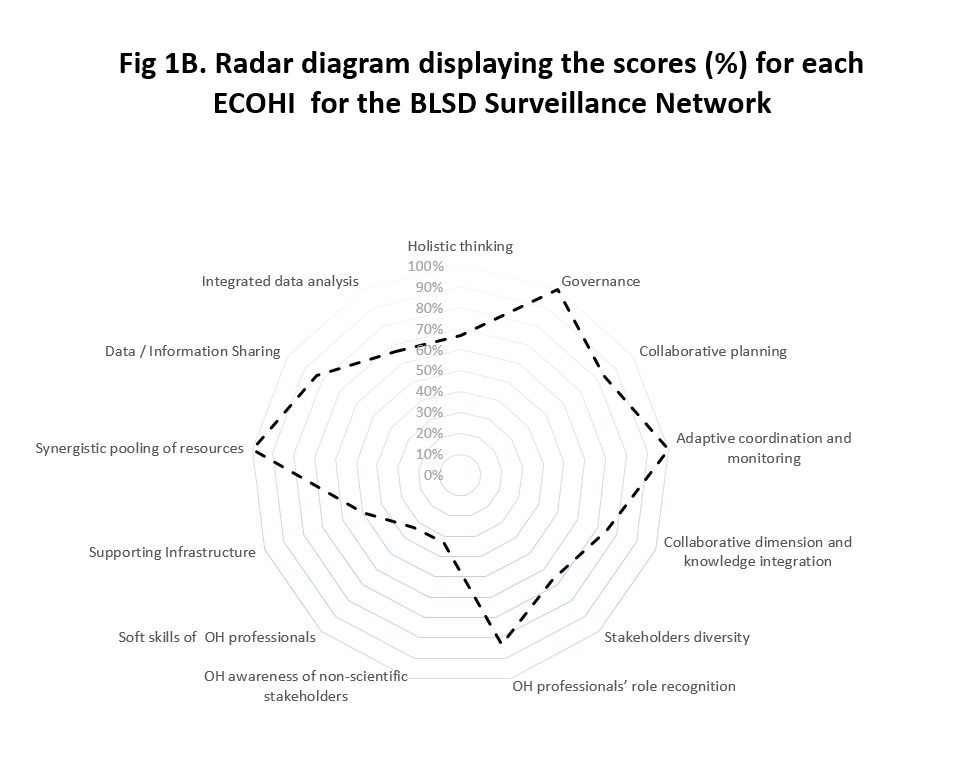

Supplement: Supplementary file 3 [file Image_2.jpg]

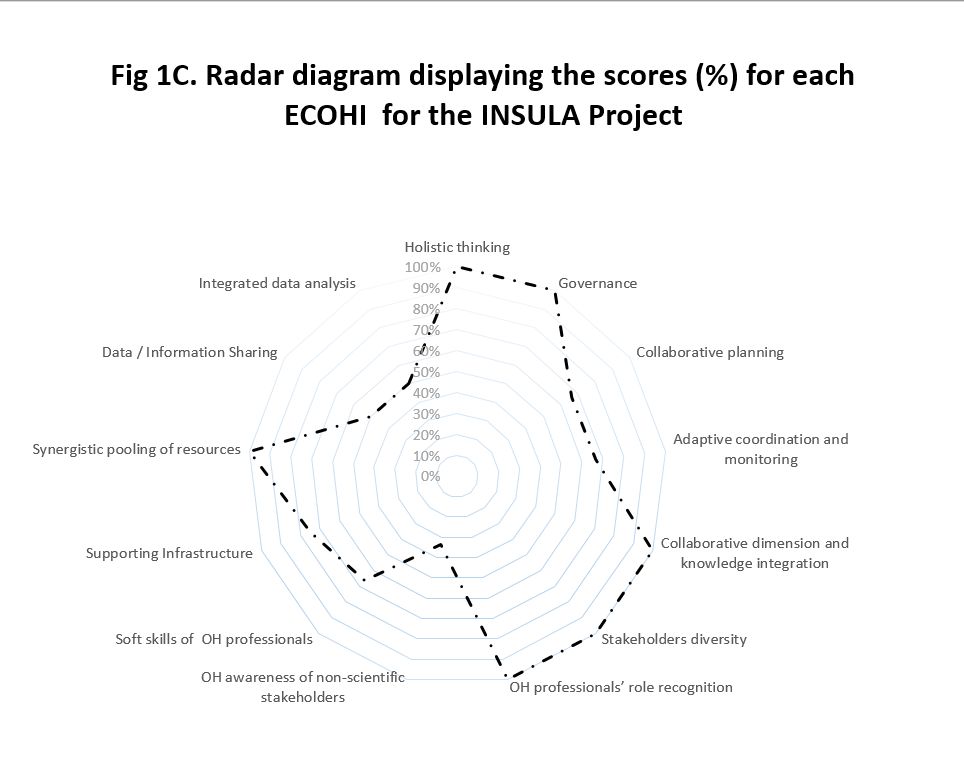

Supplement: Supplementary file 4 [file Image_3.jpg]

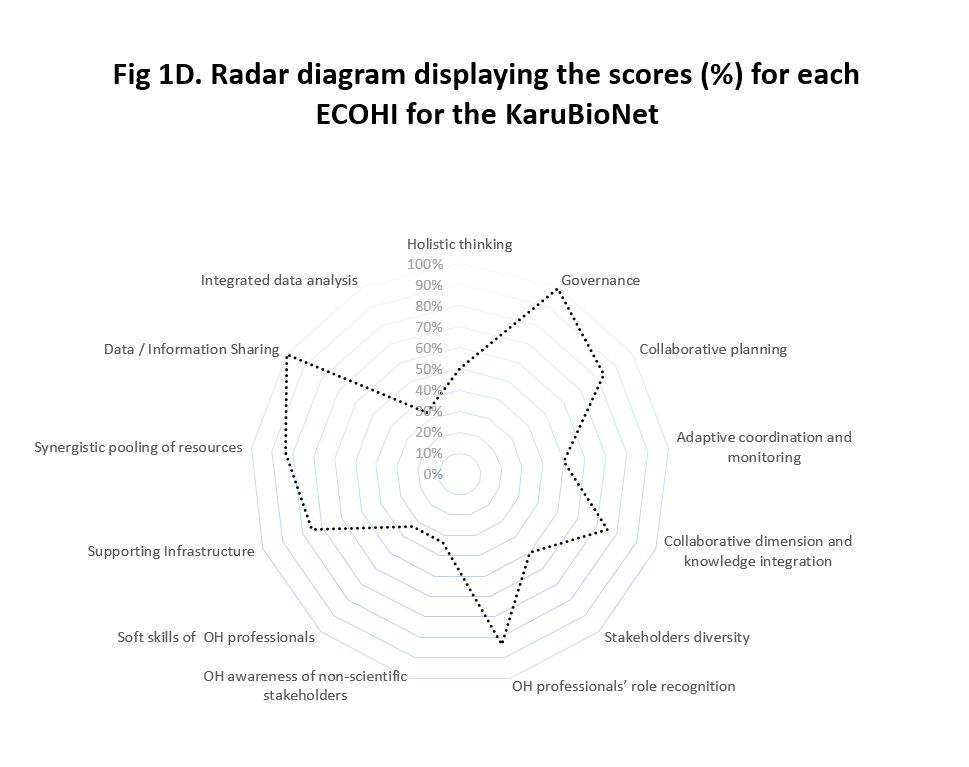

Supplement: Supplementary file 5 [file Image_4.jpg]
